# Structure-Based Virtual Screening of Natural Product-Derived Inhibitors Targeting Rv3806c in the Decaprenylphosphoryl-d-Arabinose Biosynthetic Pathway of *Mycobacterium tuberculosis*

**DOI:** 10.3390/ijms27125258

**Published:** 2026-06-10

**Authors:** Muhammad Ibrash Khan, Irfa Asghar, Bedur Faleh Ali Albalawi, Allah Ditta, Samavia Akhter, Muhammad Tayyab, Syed Basit Shah, Usama Bin Asghar, Maria Kanwal Ali, Sajid Ali, Manan Khan, Muhammad Imtiaz, Muhammad Ali

**Affiliations:** 1 Department of Biotechnology, COMSATS University Islamabad, Abbottabad Campus, Abbottabad 22060, Pakistan; khanibrash9@gmail.com (M.I.K.); mtayyab78654@gmail.com (M.T.); basitshah@cuiatd.edu.pk (S.B.S.);; 2 Department of Biology, University of Tabuk, Tabuk 71491, Saudi Arabia; bdalbalawi@ut.edu.sa; 3 Department of Environmental Sciences, Shaheed Benazir Bhutto University Sheringal, Dir (U) 18000, Pakistan; allah.ditta@sbbu.edu.pk; 4 School of Biological Sciences, The University of Western Australia, Perth, WA 6009, Australia; 5 Institute of Nuclear Medicine, Oncology and Radiotherapy (INOR), Abbottabad 22060, Pakistan; maria.kanwal.ali@outlook.com; 6 Institute of Biotechnology and Microbiology, Bacha Khan University, Charsadda 24420, Pakistan; sajidjan@live.com; 7 Department of Biotechnology and Genetic Engineering, Hazara University, Mansehra 21300, Pakistan; drmanankhan@hu.edu.pk; 8 Soil and Environmental Biotechnology Division, National Institute for Biotechnology and Genetic Engineering-College (NIBGE-C), Faisalabad 38000, Pakistan; m.imtiazpk92@yahoo.com

**Keywords:** molecular docking, structure-based drug discovery, molecular dynamics simulations, binding free energy (MM-PBSA), virtual screening

## Abstract

Phosphoribosyl transferase (Rv3806c) is a key enzyme in *Mycobacterium tuberculosis*. It is involved in the biosynthesis of decaprenylphosphoryl arabinofuranose, which is the sole donor of arabinofuranose residues in the biosynthesis of arabinogalactan and lipoarabinomannan. Inhibition of Rv3806c disrupts cell wall assembly, making it an attractive target for anti-tuberculosis drug development. In this study, a structure-based computational approach was employed to find natural inhibitors of Rv3806c. In silico ADMET filtration of 36,530 compounds from the Natural Products Atlas (NPAtlas) database and 105,909 compounds from the Bioactivity of Indian Medicinal Plants (BIMP) database yielded 285 and 553 compounds, respectively. Molecular docking analysis identified four compounds (NPA004179, NPA011911, BIMP003941, and BIMP004391) with binding affinities (−8.2, −7.5, −7.8, and −8.6 kcal/mol), respectively, stronger than the binding affinity of the native ligand (−7.2 kcal/mol). Molecular dynamics simulations demonstrated that all complexes exhibited low structural deviation, consistent hydrogen bonding, and stable protein–ligand compactness throughout the simulation period. MMPBSA analysis revealed thermodynamic stability of the Rv3806c–ligand complexes with binding energies ranging from (–14.91 to –26.30 kcal/mol). These computational findings may serve as a useful starting point for further optimization and experimental validation towards anti-tuberculosis therapeutics targeting Rv3806c.

## 1. Introduction

Tuberculosis is an airborne, communicable infection, caused by a critical human pathogen, *Mycobacterium tuberculosis* [1]. It is an infection of the lungs primarily, which is commonly known as pulmonary tuberculosis [2]. It can also spread to and infect other organs of the body, resulting in extrapulmonary tuberculosis [3]. Tuberculosis has now become the leading infectious disease killer, surpassing COVID-19. About 10 million new cases were reported, and approximately 1.2 million people died of tuberculosis in 2024 alone [4]. About a quarter of the world is suffering from latent tuberculosis infection (LTBI). Approximately 5–15% of individuals with LTBI are at risk of progressing to active tuberculosis [5]. Immunocompromised patients, especially patients with Human Immuno-deficiency Virus (HIV) infection, are at greater risk of acquiring the disease [6]. About 619,000 HIV patients fell ill with tuberculosis, and about 150,000 lost their lives to tuberculosis in 2024 alone [4]. Despite being curable, tuberculosis continues to exert substantial burden in terms of morbidity and mortality worldwide, primarily due to antibiotic resistance [7].

Standard therapy for drug-susceptible tuberculosis consists of isoniazid, rifampicin, ethambutol, and pyrazinamide [8], but the emergence of multidrug-resistant (MDR) and extensively drug-resistant (XDR) strains poses a serious threat [9]. In 2024, about 390,000 people developed MDR or rifampicin-resistant tuberculosis (MDR/RR-TB) globally, yet only about 42% of them were diagnosed and treated properly [4]. For the treatment of drug-resistant strains, second-line drugs, including bedaquiline, linezolid, delamanid, pretomanid, and moxifloxacin, are used [10]. Even with newer agents, the cure rates for MDR/RR-TB are only around 71% [4]. Even more concerning is the emergence of XDR strains, which show resistance to rifampicin and isoniazid and additional resistance to fluoroquinolones and at least one priority drug such as bedaquiline or linezolid [11]. The emerging reports of resistance even to newer drugs highlight the adaptive capacity of *M. tuberculosis* and the continuous need for innovative therapeutic targets [10].

Earlier studies have shown that inhibiting the biosynthesis of the constituents of *M. tuberculosis*’s cell wall and their precursors is an impactful strategy to tackle tuberculosis [12,13]. *M. tuberculosis*’s cell wall is composed of the mycolyl-arabinogalactan–peptidoglycan (mAGP) complex, along with lipoarabinomannan and other glycolipid conjugates [14]. It not only provides structural rigidity but also forms a formidable permeability barrier, making the bacterium intrinsically resistant to antibiotics [15]. Arabinogalactan and lipoarabinomannan are among the key constituents of the mycobacterial cell wall, playing vital roles in maintaining cell wall integrity and modulating host immune recognition [16,17]. Both of them are composed of arabinan chains rich in arabinofuranose residues [18]. Decaprenylphosphoryl-d-arabinose (DPA) serves as a sole donor of d-arabinofuranose residues in the biosynthesis of arabinogalactan and lipoarabinomannan [19]. Inhibition of this DPA biosynthesis pathway disrupts cell wall formation, increases permeability, and leads to bacterial death [20]. Enzymes within the DPA pathway, including DprE1 and DprE2, have been extensively investigated as potential drug targets [21]. Inhibitors targeting DprE1 have already progressed into clinical development, providing strong validation of the DPA pathway as a druggable and therapeutically relevant metabolic route [22]. This clinical advancement supports the development of novel anti-tubercular agents by exploring other critical enzymes involved in this pathway.

Phosphoribosyl transferase (Rv3806c) is a membrane-bound enzyme that catalyzes the first committed step in the DPA biosynthetic pathway, making it crucial for cell wall biosynthesis. It catalyzes the transfer of a phosphoribosyl group from phosphoribosyl 5-pyrophosphate (PRPP) to decaprenyl phosphate, producing decaprenylphosphoryl ribose 5-phosphate (DPPR). DPPR is subsequently dephosphorylated and epimerized to form DPA [18]. A previous conditional knockout study has confirmed that Rv3806c is essential for *M. tuberculosis* survival [20]. Previous studies have also shown that mutations in Rv3806c contribute to ethambutol resistance by enhancing decaprenylphosphoryl arabinose production, which counteracts ethambutol-mediated disruption of arabinan synthesis [23,24]. This functional association with a frontline anti-tuberculosis drug underscores the enzyme’s central regulatory role in cell wall biosynthesis and resistance mechanisms. Targeting Rv3806c therefore offers a dual advantage: direct inhibition of a vital biosynthetic pathway and the potential to overcome ethambutol resistance. Moreover, its absence of human homologs minimizes the likelihood of off-target effects, further reinforcing its potential as a drug target [24].

In the present work, a structure-based drug design methodology was applied to find probable inhibitors of phosphoribosyl transferase (Rv3806c). Molecular docking was performed against the active site of the Rv3806c enzyme using natural compounds from two databases, NPAtlas and BIMP, which revealed multiple compounds with strong binding affinities and favorable interactions within the active site. Subsequently, molecular dynamics simulations were performed for 100 ns, which demonstrated stable structural stability of the protein–ligand complexes. Binding free energy analysis showed the thermodynamic stability of the protein–ligand complexes. These computational findings provide a basis for targeting Rv3806c in the development of novel therapeutics against *M. tuberculosis*.

## 2. Results

### 2.1. In Silico ADMET Analysis

**Figure 1:**
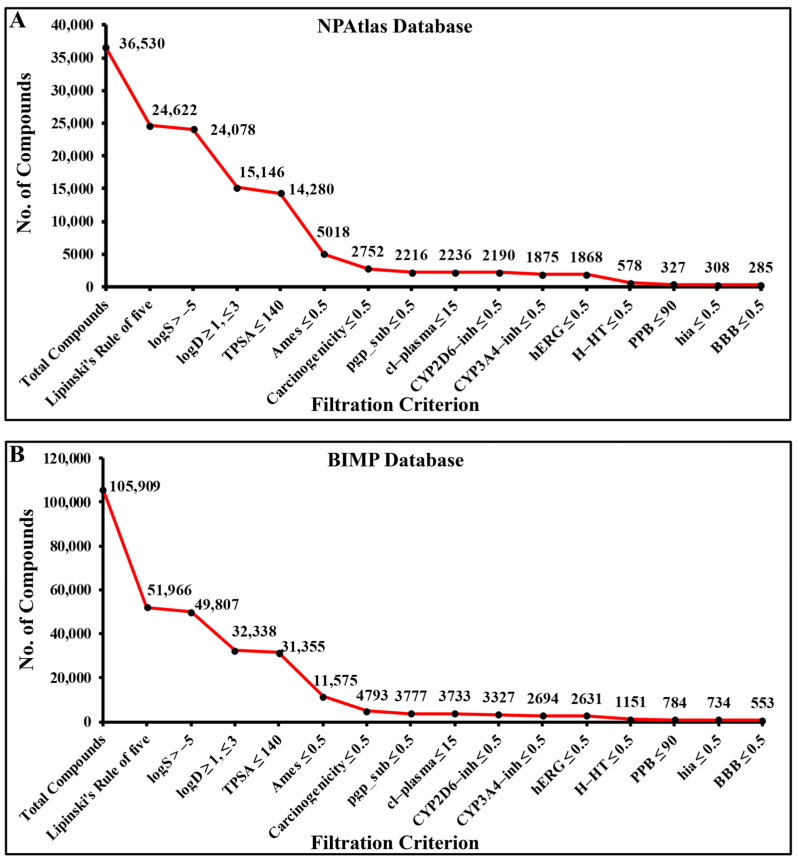
Funnel plots depicting filtration of the compounds of two databases (**A**) NPAtlas and (**B**) BIMP on the basis of their ADMET and physicochemical properties.

In silico pharmacokinetics analysis is a key step in drug design studies, as it predicts the drug-likeliness of compounds [25]. Physicochemical and ADMET-based filtration was performed on 105,909 compounds from the BIMP database and 36,350 from the NPAtlas databases. A total of 285 compounds from the NPAtlas database and 553 compounds from the BIMP database were shortlisted for subsequent molecular docking analysis against the target protein (Figure 1). Lipinski’s rule of five serves as the gold standard for virtual screening of ligands on the basis of their physicochemical properties [26]. Lipinski’s rule of five was applied, and all the compounds that had zero violations were retained. Lipophilicity of a ligand is a crucial parameter. Since cell membranes are lipophilic in nature, the drug needs to be lipophilic enough to pass through them. The ligands that had lipophilicity logP (pH-independent) and logD (pH-dependent) values in the best range for effective passive diffusion were retained. The ADMET properties of the compounds are also crucial for understanding how the drug will behave within the body. In the context of absorption, the drug must be actively absorbed from the human intestine to reach plasma and then be distributed to the target tissue [27]. All ligands that had acceptable human intestinal absorption (hia) values were retained. Another important factor that may hinder the absorption process is the presence of P-glycoprotein receptors on the cell membrane. They actively pump drugs out of cells, thus reducing drug concentrations within cells [28]. Non-substrates of P-glycoprotein were retained.

Solubility (LogS) (≥−10, ≤0.5), pH-dependent lipophilicity (LogD) (≤3, ≥1), Total Polar Surface Area (TPSA) (≤140), Ames (≤0.5), Carcinogenicity (≤0.5), protein glycoprotein substrate (pgp_sub) (≤0.5), plasma clearance (cl-Plasma) (>0, ≤15), cytochrome isoform CYP2D6 inhibitor (≤0.5; non-inhibitor), cytochrome isoform CYP3A4 inhibitor (≤0.5; non-inhibitor), human-ether-a-go-go-related gene (hERG) (≤0.5), human liver toxicity (≤0.5), plasma protein binding (PPB) (≤90), human intestinal absorption (hia) (≤0.5), and blood–brain barrier (BBB) (≤0.5).

After absorption, the drug needs to be distributed to the target tissues. Apart from the physicochemical properties of the drug, one thing that hinders this distribution is the plasma protein binding. There are proteins, like albumin, in the plasma to which drugs may bind, and because of this, the amount of free drug available for distribution to the tissues within the plasma drops [29]. All the ligands that had plasma protein binding affinity less than 90% were retained. Most drugs that work in the central nervous system require permeability through the blood–brain barrier. The blood–brain barrier permeators were retained. Once absorbed, the drugs also need to be metabolized; otherwise, they can accumulate within tissues and cause adverse effects. CYP450 enzyme isoforms present in the body metabolize drugs to either an inactive form or a less toxic form and sometimes activate them as prodrugs [30]. It needs to be verified that the proposed ligand is not an inhibitor of these enzymes, which might result in drug–drug interactions. This is particularly relevant in tuberculosis patients with HIV co-infection, where polypharmacy is common and treatment-induced toxicities are a major concern [31]. CYP2D6 and CYP3A4 isoforms metabolize more than 50% of drugs [32]. Non-inhibitors of these enzymes were retained. Cl-plasma is an important factor in the context of excretion of a drug. Cl-plasma is the concentration of the plasma that is completely cleared of a drug in unit time. This is important in determining the dosage concentration and also the interval after which the next dosage can be given [33]. All ligands that fulfilled the criteria were kept. The toxicity profiles of a drug must be checked before it is used as a therapeutic. The human ether-a-go-go-related gene (hERG) encodes a potassium channel protein required for the heart’s normal activity. If this protein channel gets blocked, it results in QT prolongation, which can result in sudden death [34]. All non-blockers of hERG were retained. Ames is another test used to check the mutagenicity of compounds [35]. Non-mutagenic ligands were retained. Only non-carcinogenic ligands were retained. The liver is an important organ of the body, especially in the context of detoxification of potentially harmful substances. Some drugs are toxic to the liver, which may result in serious complications [36]. All the non-hepatotoxic ligands were retained.

### 2.2. Molecular Docking Analysis

**Figure 2:**
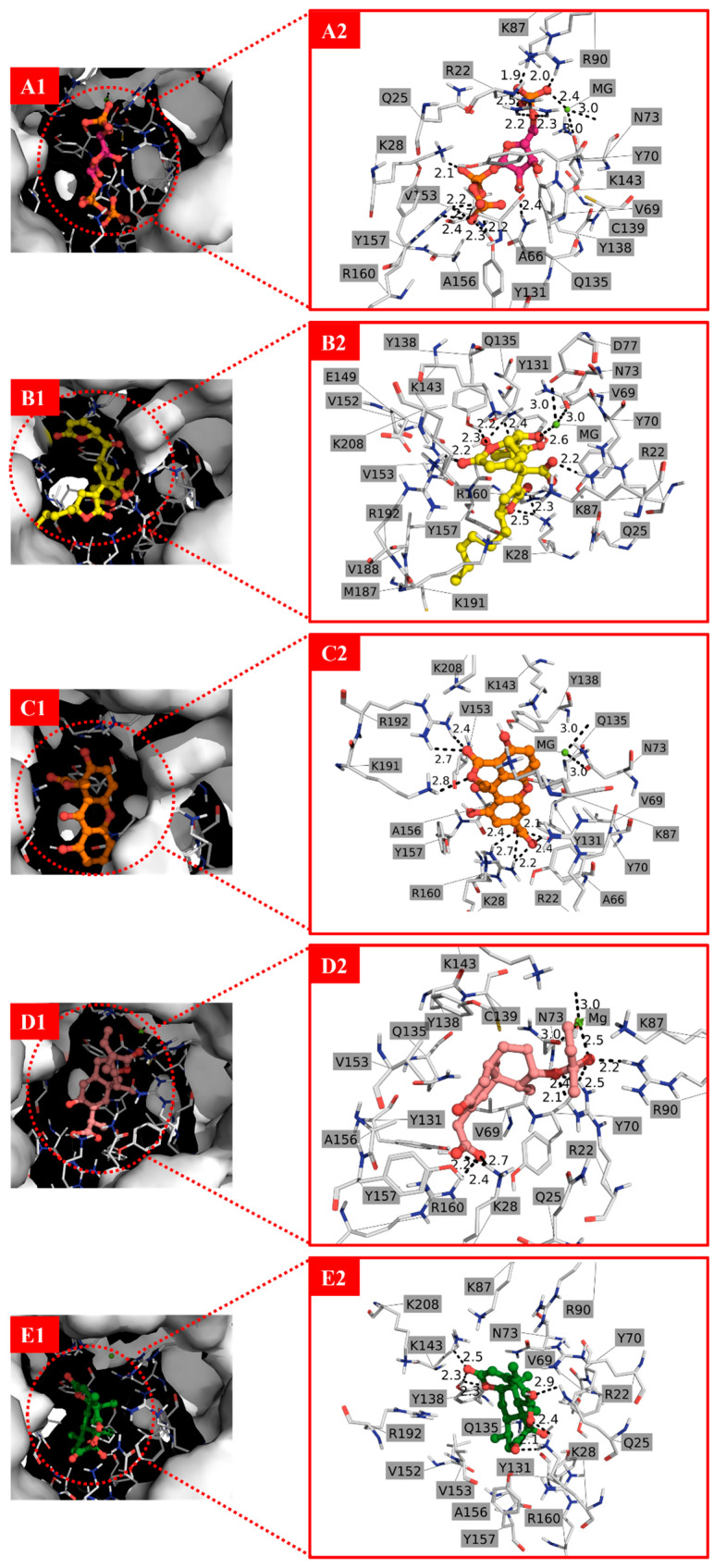
Schematic diagram showing binding poses and interaction profiles of the native ligand and four putative ligands with Rv3806c. Binding poses (**A1**) native ligand (purple), (**B1**) NPA004179 (yellow), (**C1**) NPA011911 (orange), (**D1**) BIMP003491 (pink), and (**E1**) BIMP004391 (green) are shown in surface representation. Close-up representations providing insight into the amino acid residues forming binding pockets and binding interactions between the Rv3806c–ligand complexes are shown in panels (**A2**), (**B2**), (**C2**), (**D2**), and (**E2**), respectively. Polar contacts along with their corresponding distances are indicated using black dashed lines.

Molecular docking was conducted between target protein Rv3806c and 285 and 553 compounds of the NPAtlas and BIMP databases, respectively. The molecular docking of the native substrate phosphoribosyl 5-pyrophosphate was also performed with the target protein Rv3806c. The binding affinity of the best pose of the native substrate was (−7.2 kcal/mole). This energy value was set as the threshold for the selection of putative ligands. The ligands that had better binding energies than that of the native substrate were analyzed for their binding interactions within the active site of Rv3806c. As a result, four compounds were shortlisted as promising candidates. Schematic diagrams of the binding poses and interactions of the four putative ligands in complex with the target protein are shown in (Figure 2).

Ligand NPA004179 had six hydrogen bond interactions, out of which five residues, ARG22 (2.2 Å), LYS28 (2.5 Å), ASN73 (3.0 Å), GLN135 (2.4 Å) and TYR138 (2.3 Å), were also involved in stabilizing the native substrate within the binding pocket of Rv3806c (Figure 2(B2). The ligand also had three alkyl, pi–alkyl interactions, one of them with LYS191. Along with it, metal-acceptor bonds and 10 van der Waals interactions were also formed, out of which four interactions were with key amino acids TYR70, LYS87, LYS143 and TYR157. Ligand NPA011911 had four hydrogen bonds, three of which were with residues TYR131 (2.4 Å), ARG160 (2.2 Å), and LYS191 (2.8 Å), which were also involved in interactions with the native substrate (Figure 2(C2)). It also had a pi–pi interaction with residue ALA156, a pi–cation interaction with residue LYS28 and a pi–donor hydrogen bond with residue TYR138. Eight van der Waals interactions were also present, five of them with key amino acid residues TYR70, ASN73, GLN135, LYS143 and TYR157. Ligand BIMP003941 formed three hydrogen bonds with LYS28 (2.7 Å), ARG90 (2.2 Å), ARG160 (2.4 Å) (Figure 2(D2)). All three amino acids were also involved in native substrate binding and stabilization within the binding pocket of Rv3806c. It also had five alkyl and pi–alkyl interactions with five amino acids; two of them, TYR70 and TYR131, were key amino acid residues. It also had eight van der Waals interactions with Rv3806c. Six of these amino acids were also involved in the binding interactions with the native substrate. The fourth ligand, BIMP004391, had five hydrogen bonds with key amino acids of Rv3806c, including two hydrogen bonds with LYS28 (2.1 Å and 2.4 Å), two with TYR138 (2.3 Å and 2.3 Å) and one with LYS143 (2.5 Å) (Figure 2(E2)). Additionally, it formed alkyl, pi–alkyl and metal-acceptor bonds. It also had eight van der Waals interactions, out of which six residues, namely, ARG22, ASN73, ARG90, GLN135, TYR157, and ARG160, were also involved in interactions with the native substrate. The binding interactions of the probable four ligands with the amino acid residues of the target protein Rv3806c are illustrated in Table 1.

### 2.3. Selection of Probable Ligands

On the basis of drug-likeliness, binding affinity with the target protein and number of interactions with the active site residues of the target protein, the four best ligands were shortlisted, which were further processed with molecular dynamics simulation analysis. The drug-likeliness profiles of the probable ligands are provided in Table 2.

The complete data of the four best ligands with their IUPAC names, 2D structures, binding affinities obtained through molecular docking and number of interactions with the active site amino acid residues of the target enzyme Rv3806c are provided in (Table 3).

### 2.4. Evaluation of Structural Stability of Protein–Ligand Complexes

**Figure 3:**
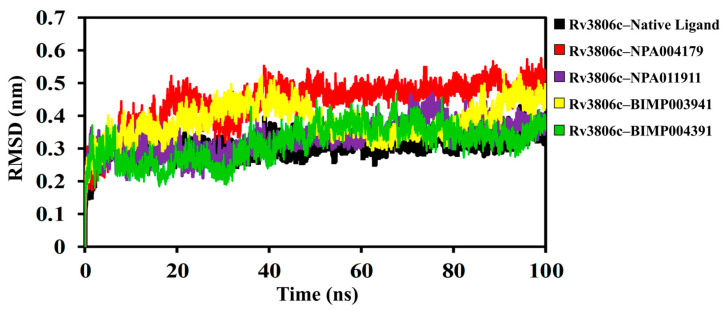
Root Mean Square Deviation (RMSD) plot showing the average displacements of the backbone atoms of Rv3806c when bound to the native ligand and four probable ligands. Low RMSD values of the protein–ligand complexes indicate stable binding during the 100 ns simulation.

Root Mean Square Deviation (RMSD) is a vital indicator for assessing the average displacements of the atoms of the protein–ligand complexes relative to their reference docked structures over the simulation period. The RMSD values of the protein–ligand complexes were calculated using the backbone atoms of the protein to assess structural stability and overall conformational deviations during the 100 ns simulation period (Figure 3).

Among the five complexes, the native ligand bound to the target protein provided the best stability, with an average RMSD value of 0.3117 ± 0.034264 nm. Its trajectory remained stable within the 0.25–0.35 nm range. Ligand NPA011911 and ligand BIMP004391 had almost the same trajectories as that of the native ligand. They had average RMSD values of 0.33029 ± 0.0472 nm and 0.3167 ± 0.0554 nm, respectively. Ligand NPA004179 showed the highest deviation among the four complexes, which had an average RMSD value of 0.4431 ± 0.0708 nm. It showed an initial rise in trajectory up to 0.5 nm and then remained stable between 0.4 and 0.5 nm. The average RMSD values computed using the respective trajectories of protein–ligand complexes are listed in (Table 4).

### 2.5. Evaluation of Residual Fluctuations in Protein–Ligand Complexes

**Figure 4:**
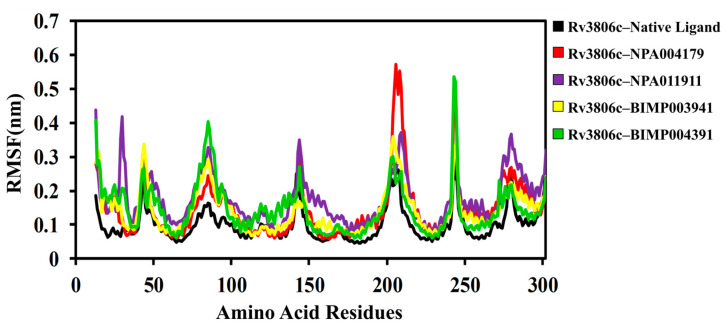
Root Mean Square Fluctuation (RMSF) plot presenting the per-residue flexibility of the Rv3806c protein when bound to the native ligand and four probable ligands. All complexes exhibited RMSF values less than 0.2 nm, which indicates the stabilization of the target protein Rv3806c upon ligand binding.

Root Mean Square Fluctuation (RMSF) analysis of the MD trajectories measures how much each amino acid in the protein fluctuates during the simulation, giving a clear picture of which regions are flexible and which remain relatively stable over time. Among the different protein–ligand complexes, the one with the native ligand showed the least fluctuation, with an average RMSF value of 0.1002 ± 0.0498 nm, which indicated a strong stabilizing effect on the protein (Figure 4). The complexes with ligands NPA004179, BIMP003941 and BIMP004391 demonstrated slightly more residual flexibility, with average RMSF values of 0.1417 ± 0.0864 nm, 0.1408 ± 0.0629 nm, and 0.1473 ± 0.0742 nm, respectively. Meanwhile, the RMSF trajectory for NPA011911 exhibited the most residual fluctuations, with an average value of 0.1758 ± 0.0720 nm.

### 2.6. Evaluation of Structural Compactness of Protein–Ligand Complexes

The radius of gyration (Rg) essentially describes compactness or expansion of the protein structure during a simulation. It is a useful measure for evaluating whether a protein stays tightly folded or starts to loosen up under the influence of different ligands.

**Figure 5:**
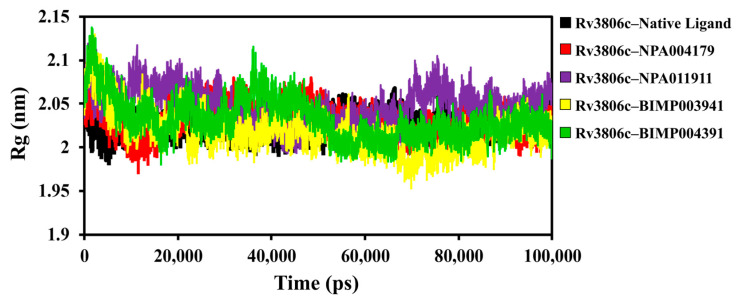
Radius of gyration (Rg) analysis illustrating the overall compactness and conformational stability of Rv3806c during 100 ns molecular dynamics simulations when bound with five different ligands. Rg values remaining consistent, typically within a narrow range of (1.96 to 2.14 nm), indicate stable protein folding and minimal structural deviations throughout the simulation.

Across the 100 ns simulation, all five complexes showed fairly consistent Rg values, fluctuating within a narrow range of 1.96 to 2.14 nm (Figure 5). This consistency suggested that the protein remained structurally stable and did not undergo any major unfolding or collapse. Among all the ligands, the complex with ligand BIMP003941 maintained the lowest and steadiest Rg values, with an average Rg value of 2.0176 ± 0.0231 nm, indicating that it helped the protein stay in its most compact and stable form. The complex with the native ligand had an average Rg value of 2.0258 ± 0.0170 nm, which pointed to its good stabilizing effect on the protein structure. On the other hand, the complexes with NPA004179, NPA011911 and BIMP004391 showed slightly higher Rg values of 2.0303 ± 0.0168 nm, 2.04792 ± 0.0204 nm, and 2.0326 ± 0.0237 nm, respectively, with more noticeable fluctuations, especially at the beginning and toward the end of the simulation. This meant that these ligands introduced a bit more flexibility or looseness in the overall protein structure. However, it is important to note that none of the complexes exhibited drastic Rg changes, which confirmed that the protein remained properly folded and dynamically stable in all cases.

### 2.7. Evaluation of Hydrogen Bonds of Protein–Ligand Complexes

**Figure 6:**
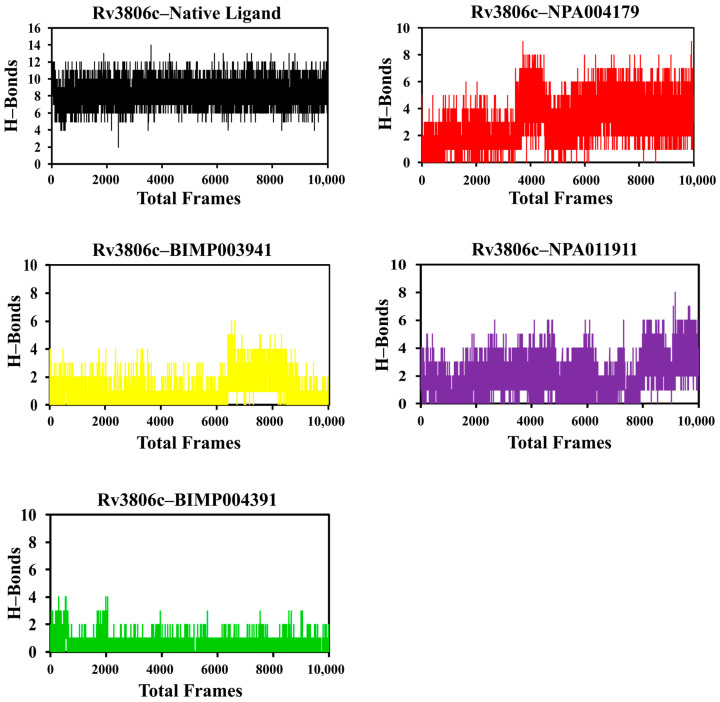
Analysis of hydrogen bond interactions between proteins and ligands during a 100 ns simulation run. Native ligand (black), ligand NPA004179 (red), ligand NPA011911 (purple), ligand BIMP003941 (yellow) and ligand BIMP004391 (green).

Hydrogen bond analysis was performed to find the degree of interaction between the ligands and Rv3806c (Figure 6). The native ligand formed consistent six to 12 hydrogen bonds with the target protein. Among the screened ligands, ligand NPA004179 formed two to six hydrogen bonds, thus showing good stability. At the starting frames of the simulation, it showed fluctuations, but in the last 5000 frames, it consistently formed six hydrogen bonds, which suggests that it found a good conformation within the binding site after the first 5000 frames. Ligand NPA011911 formed one to five hydrogen bonds across the simulation period, showing a relatively stable interaction with Rv3806c. Ligands BIMP003491 and BIMP004391 showed only two consistent hydrogen bonds with Rv3806c.

### 2.8. Binding Interactions Analysis

**Figure 7:**
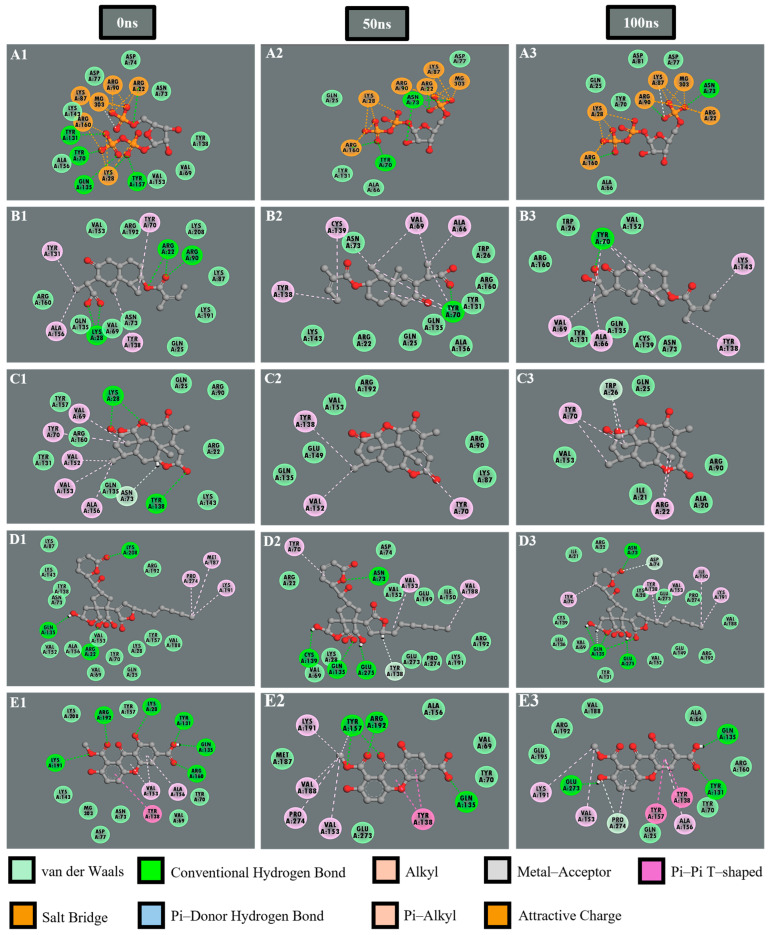
2D diagrams depicting the binding interaction profiles of native ligand (**A1**–**A3**), BIMP003941 (**B1**–**B3**), BIMP004391 (**C1**–**C3**), NPA004179 (**D1**–**D3**), and NPA011911 (**E1**–**E3**) with amino acid residues of Rv3806c, at time frames: 0 ns (**A1**–**E1**), 50 ns (**A2**–**E2**), and 100 ns (**A3**–**E3**). Legends at the bottom of the figure indicate the color coding of bonds, where each color represents a specific type of interaction.

Analysis of the 2D interaction profiles of the Rv3806c–ligand complexes at 0, 50, and 100 ns was performed (Figure 7). The native ligand formed hydrogen bonds, salt bridges, and van der Waals contacts with residues such as ARG22, LYS28, LYS87, ARG90, ARG160, and MG303, playing key roles in stabilizing the complex (Figure 7(A1–A3)).

Among the screened ligands, NPA004179 displayed stable binding behavior, consistently forming hydrogen bonds and hydrophobic interactions with key residues TYR70, ASN73, GLN135, and TYR138 at various time frames during the simulation (Figure 7(D1–D3)). Similarly, NPA011911 maintained persistent polar and nonpolar contacts with essential residues GLN135, TYR138, VAL153, TYR157, and LYS191, many of which were also involved in stabilizing the native ligand (Figure 7(E1–E3)). These sustained polar and nonpolar interactions suggest that both ligands fit well within the active site, supporting strong binding stability. In contrast, BIMP003941 formed fewer hydrogen bonds but showed steady hydrophobic contacts with residues VAL69, TYR70, and TYR138 that supported structural integrity (Figure 7(B1–B3)), while BIMP004391 showed a gradual decline in both hydrogen bonding and hydrophobic interactions, indicating moderate conformational flexibility and comparatively weaker binding stability, as shown in Figure 7(C1–C3).

### 2.9. Binding Free Energy Analysis

The binding free energy was calculated for all the protein–ligand complexes through Molecular Mechanics Poisson–Boltzmann Surface Area (MM-PBSA) using the gmx_MMPBSA package (Table 5). The average binding free energy (ΔG_bind_) of the native ligand–protein complex was (−180.07 ± 0.38 kcal/mol), which implies a thermodynamically stable formation of the complex. The average electrostatic energy (ΔG_elec_) (−1904.02 ± 1.24 kcal/mol) contributed the most to the stability of the complex. The average van der Waals energy (ΔG_vdW_) (16.75 ± 0.21 kcal/mol) and polar solvation energy (ΔG_polar_) (1710.14 ± 1.09 kcal/mol) opposed the binding. The average nonpolar solvation energy (ΔG_non-polar_ or ΔG_SASA_) (−2.95 ± 0.01 kcal/mol), derived through solvent-accessible surface area (SASA), stabilized the complex. The ligand NPA004197-RV3806c complex had an average binding energy of (−17.49 ± 0.2 kcal/mol). The ΔG_vdW_ (−52.78 ± 0.1 kcal/mol) along with ΔG_elec_ (−37.02 ± 0.24 kcal/mol) provided stability to the complex. ΔG_polar_ (77.56 ± 0.33 kcal/mol) opposed the formation of the complex, while ΔG_non-polar_ or ΔG_SASA_ (−5.25 ± 0 kcal/mol) stabilized the complex. The ligand NPA011911-RV3806c complex had an average binding energy of (−26.3 ± 0.15 kcal/mol). ΔG_vdW_ (−30.3 ± 0.1 kcal/mol) along with ΔG_elec_ (−53.58 ± 0.31 kcal/mol) provided stability to the complex. ΔG_polar_ (61.29 ± 0.22 kcal/mol) opposed the formation of the complex, while ΔG_non-polar_ or ΔG_SASA_ (−3.71 ± 0 kcal/mol) stabilized the complex. The BIMP003941-Rv3806c and BIMP00439-Rv3806c complexes exhibited binding free energies of (−21.94 ± 0.12 kcal/mol) and (−14.91 ± 0.09 kcal/mol), respectively. ΔG_vdW_ (−36.77 ± 0.07 kcal/mol) and (−26.45 ± 0.07 kcal/mol), respectively, remained the major contributor to their stabilities. ΔG_elec_ (−2.61 ± 0.19 kcal/mol) and (−3.73 ± 0.24 kcal/mol), respectively, favored the formation of the complexes. ΔG_polar_ (21.33 ± 0.15 kcal/mol) and (18.06 ± 0.22 kcal/mol), respectively, opposed the formation of the complexes, while ΔG_SASA_ (−3.89 ± 0 kcal/mol) (2.8 ± 0.01 kcal/mol), respectively, stabilized the complexes.

## 3. Discussion

The decaprenylphosphoryl-D-arabinose (DPA) biosynthesis pathway is essential for the survival of *M. tuberculosis*, as it is the only arabinofuranose donor needed for the formation of the arabinogalactan and lipoarabinomannan components of the cell wall [19,20]. Consequently, enzymes involved in its biosynthetic pathway are considered as potential therapeutic targets. Drugs have already progressed into clinical trials against DprE1, an enzyme involved in the DPA biosynthetic pathway [22]. Phosphoribosyl transferase (Rv3806c) is another critical enzyme involved in the biosynthesis of DPA. Although Rv3806c has been genetically confirmed to be essential for DPA biosynthesis [20], it has not yet been actively pursued as a target for drug development, to our knowledge. The reason for this gap might be the lack of its detailed structural and mechanistic understanding. However, a recent publication of its high-resolution cryo-electron microscopy structure has offered comprehensive insights into its substrate-binding architecture and the spatial arrangement of key active site residues [24]. This structural advancement now enables rational, structure-guided exploration of Rv3806c as a potential anti-tubercular drug target.

In the current study, compounds from two databases, BIMP and NPAtlas, were screened for their ADMET and physicochemical properties. The criteria for the filtration were consistent with earlier Computer-Aided Drug Design (CADD) studies [37,38,39,40]. The compounds that fulfilled the drug-likeliness criteria were then docked to the active site of the target protein Rv3806c. Based on the binding affinities and binding interactions with the key active site residues, molecular docking revealed four compounds: NPA004179, NPA011911, BIMP003941, and BIMP004391. These compounds showed docking scores ranging from (−8.6 to −7.5 kcal/mol), surpassing the binding affinity of the native substrate (−7.2 kcal/mol). The dynamic stability of the protein–ligand complexes was further confirmed through 100 ns MD simulations. Three out of the four proposed ligands showed an average RMSD value (<0.35 nm), while ligand NPA004197 showed a slight initial rise in trajectory to (0.4 nm) and then remained stable between (0.4 and 0.5 nm). These findings are consistent with earlier studies, showing minimal conformational drift and maintenance of structural integrity throughout the simulation period [37,40]. All the protein–ligand complexes exhibited low RMSF values (<0.2 nm), indicating minimal residual fluctuations. However, noticeable peaks were observed at residue positions 45, 86, 144, 206, 243, and 275 (Figure 4). These residues lie in the cytoplasmic and periplasmic loop regions that naturally experience more fluctuations. On the other hand, the transmembrane domains that contain most of the active site residues exhibited much lower fluctuations, indicating that ligand binding helped stabilize these critical areas. These findings are in line with previous studies [40,41], implying that ligand binding helped stabilize the functionally important regions of the protein. Additionally, compact radius of gyration (Rg) profiles indicated preserved protein folding and overall structural compactness. Moreover, consistent formation of hydrogen bonds between the protein–ligand complexes indicated stable and sustained binding interactions over time, which is completely in line with previous studies [40,41].

MMPBSA binding free energy analysis revealed that all the protein–ligand complexes exhibited negative values (−14.91 to −26.3 kcal/mol), which indicates that the complex formation is thermodynamically favorable. A markedly large negative binding free energy (−180 kcal/mol) for the native substrate underscores its strong and stable accommodation within the Rv3806c binding pocket. Our findings suggest that this stability primarily arises from its coordination with the Mg^2+^ cofactor. When the MD trajectory of the native ligand–Rv3806c complex was analyzed without Mg^2+^, a positive binding free energy (+31.05 ± 2.14 kcal/mol) was observed (Figure S1F), indicating that complex formation would require energy input rather than occurring spontaneously [42]. This observation aligns with a recent structural study demonstrating that Mg^2+^ is essential for phosphoribosyl pyrophosphate binding in Rv3806c [24].

Binding interaction analysis of trajectories revealed that the proposed ligands consistently engaged residues that have been found to be critical for substrate stabilization and the catalytic mechanism of Rv3806c. Notably, the compounds formed stable hydrogen bonds and electrostatic and hydrophobic interactions with Tyr70, Asn73, Asp77, Gln135, Tyr138, and LYS191. These residues have been demonstrated to play essential roles in native substrate binding, Mg^2+^ coordination, pyrophosphate stabilization, and positioning of the ribose moiety for nucleophilic attack during phosphoribosyl transfer [24]. The interactions of the proposed ligands with Asp77 and Asn73, key residues involved in Mg^2+^-mediated stabilization of the pyrophosphate group, indicate that the predicted ligands may interfere with proper donor substrate positioning. Engagement of Gln135 and Tyr70, which contribute to ribose orientation and transition-state stabilization, further supports the likelihood of catalytic disruption. Importantly, contacts with Tyr138, a conserved residue across mycobacteria and functionally implicated in catalysis, reinforce the potential of our compounds to inhibit the catalytic mechanism of Rv3806c [24]. Collectively, the convergence of the binding interactions of the proposed ligands with residues experimentally validated as essential for substrate binding, Mg^2+^ coordination, and transition-state stabilization provides strong structural evidence that the identified compounds are positioned to interfere directly with the catalytic machinery of Rv3806c.

Furthermore, the natural origin of the shortlisted compounds strengthens their relevance as biologically meaningful scaffolds for anti-tubercular drug discovery. Proposed compounds NPA004179 and NPA011911 were sourced from *Penicillium dangeardii* Pitt and *P. citrinum*, respectively [43,44]. Filamentous fungi of the genus *Penicillium* are well known for producing structurally diverse secondary metabolites with antimicrobial, cytotoxic, and enzyme-inhibitory properties [45,46,47]. In particular, *P. citrinum* is known to produce structurally diverse polyketides and alkaloids, including citrinin derivatives with reported antibacterial activity [48]. Notably, NPA011911 belongs to the xanthone class of natural products, whose members have been extensively reported to exhibit anti-mycobacterial activity [49]. Proposed compounds BIMP003941 and BIMP004391 were sourced from *Farfugium japonicum* and *Quassia amara,* respectively. These traditional medicinal plants are rich sources of phenolics, sesquiterpenoids, and quassinoids with well-documented antioxidant, anti-inflammatory, antimicrobial, antimalarial, and anticancer activities [50,51,52,53,54]. Although the proposed compounds have not been previously reported for therapeutic use, the well-established pharmacological potential of other metabolites derived from these sources may support the probability that the screened compounds are candidates for further investigation.

While the computational analyses performed in this study provide insights into the possible binding behavior and interaction stability of the identified compounds, the absence of experimental validation remains an important limitation. Therefore, additional biochemical and structural studies will be necessary to verify the biological relevance of these findings and to further evaluate the therapeutic potential of the identified compounds against *M. tuberculosis*. Future work should focus on isolating and purifying the identified compounds, followed by enzyme inhibition assays using recombinant Rv3806c to confirm their activity. Antimicrobial testing against *Mycobacterium tuberculosis* strains, alongside cytotoxicity evaluation in mammalian cells, will be essential to assess therapeutic potential and safety. These steps may prove crucial for bridging computational predictions with experimental validation, ultimately advancing our findings toward viable therapeutic candidates.

## 4. Materials and Methods

### 4.1. Protein Retrieval and Optimization

The structure of enzyme Rv3806c bound with its native substrate, phosphoribosyl 5-pyrophosphate (PDB ID: 8J8J), predicted through electron microscopy with a resolution of 2.76 (Å), was downloaded from the Protein Data Bank [24]. The downloaded protein needed to be optimized for molecular docking analysis. AutoDock tools (version 1.5.7) was used for this purpose [55]. All the extra chains and hetero atoms were removed, except a Mg^2+^ ion, which is a cofactor for the enzyme. Polar hydrogen and Kollman charges were added, and it was ensured that the charges were spread on all the residues. This modified structure was then saved as a receptor (pdbqt) file.

### 4.2. Active Site of Rv3806c

The active site residue data was retrieved from Uniprot and also by using the 2D interaction diagram of the native ligand phosphoribosyl 5-pyrophosphate bound to Rv3806c (PDB ID: 8J8J), using Biovia Discovery studio [24,56,57]. The active site amino acid residues were: ARG22, LYS28, TYR70, ASN73, LYS87, ARG90, TYR131, GLN135, TYR138, LYS143, VAL153, ALA156, ARG160 and TYR191 (https://www.uniprot.org/uniprotkb/P9WFR5/entry, accessed on 17 January 2025).

### 4.3. Ligand Library Construction

Two databases, BIMP (https://scfbio.iitd.ac.in, accessed on 18 January 2025) and NPAtlas [58], consisting of natural compounds, were selected. A total of 105,909 phytochemicals derived from 6209 medicinal plants were retrieved from the BIMP database. The complete dataset of 36,530 compounds was retrieved from the NPAtlas database (version 2024_09), which is composed of natural compounds derived from bacteria and fungi.

The downloaded compounds were subjected to filtration on the basis of physicochemical properties and ADMET (Absorption, Distribution, Metabolism, Excretion, Toxicity) profiles using an online tool, ADMETLab 3.0 [59]. The resulting compounds were then prepared for molecular docking by minimizing their energies and converting them to (pdbqt) formats using Openbable (version 3.1.1) [60].

### 4.4. Molecular Docking

Molecular docking is a computational technique used to determine how ligands will interact with target proteins [61]. The prepared ligand library was then docked with the prepared protein using AutoDock Vina (version 1.2.0), which is known for its efficiency, speed and accuracy in predicting binding affinities [62]. A grid box with a center at x = 150.874, y = 177.146, and z = 133.700 and dimensions of 40 Å × 66 Å × 76 Å was defined to accommodate all the active site residues of the target protein Rv3806c. The exhaustiveness was set to 16, while the number of generated poses was kept at the default setting.

Prior to molecular docking, complexes of the protein with the best poses of ligands were made using the PyMOL visualization tool (version 3.1) [63]. The interactions of the ligands with the active site amino acids were compared to the interaction profile of the enzyme’s native substrate using 2D interaction diagrams, obtained through Biovia Discovery Studio (version 21.1.0.20298). Ligands with minimum binding affinities and maximum interactions with active site amino acids were shortlisted for molecular dynamics simulation analysis.

### 4.5. Molecular Dynamics Simulations

For molecular dynamics simulations, Groningen Machine for Chemical Simulations (GROMACS) version 2023.3 was used [64]. The best poses of the protein–ligand complexes obtained through molecular docking were used for molecular dynamics simulations. The force field Chemistry at Harvard Macromolecular Mechanics36 (CHARMM36), along with the TIP3P water model, was used [65]. SwissParam (https://www.swissparam.ch/, accessed on 25 May 2025) was used to generate ligand parameter files [66]. Na^+^ and Cl^−^ ions were added to neutralize the charge in the system. The equilibration of the system was performed using the NVT ensemble for 100 ps to stabilize the temperature at 300 K with the V-rescale thermostat [67], followed by pressure equilibration through the NPT ensemble to stabilize the pressure at 1 bar using the Parrinello–Rahman barostat [68]. The production run was then performed for 100 ns using a 2 femtoseconds (fs) time step with the leap-frog integrator, and system snapshots were saved every 10 ps. Following the 100 ns production run, the resulting trajectories were examined to evaluate the structural stability and interaction dynamics of the protein–ligand complexes.

### 4.6. MM-PBSA Analysis

To estimate the binding free energies of the protein–ligand complexes, the Molecular Mechanics Poisson–Boltzmann Surface Area (MM-PBSA) approach was applied using the gmx_MMPBSA package [69,70]. Binding free energies of all the protein–ligand complexes were calculated using the last 1000 frames from their respective MD trajectories. The binding free energies of the protein–ligand complexes were determined by using the following equation:

ΔG_bind_ = G_complex_ − (G_protein_ + G_ligand_)

where ΔG_bind_ represents the total binding free energy, G_complex_ represents the energy of the protein and ligand when bound in a complex, and G_protein_ and G_ligand_ represent the energies of the protein and ligand, respectively, in an unbound state.

## 5. Conclusions

Tuberculosis is a treatable disease, yet it continues to cause millions of deaths each year, largely due to the growing threat of antibiotic resistance among *M. tuberculosis* strains. This highlights the urgent need to explore novel drug targets and therapeutic agents. In the present study, Rv3806c, an underexplored enzyme involved in the decaprenylphosphoryl-d-arabinose biosynthetic pathway, was investigated through a computational approach. The findings provide preliminary insights into the interaction patterns and binding stabilities of the screened compounds against Rv3806c and support the broader potential of targeting relatively unexplored enzymes in *M. tuberculosis*. However, the present study is entirely computational and, therefore, experimental validation through enzyme inhibition assays, MIC testing, and cytotoxicity screening is essential to confirm the inhibitory potential and therapeutic applicability against *M. tuberculosis*.

## Figures and Tables

**Table 1:** Key binding interactions between probable ligands and amino acid residues of Rv3806c obtained from 2D interaction diagrams.

Compound_ID	Amino Acid Residues of Rv3806c Involved in Binding Interactions				
	Hydrogen Bonds	Salt Bridges	Hydrophobic Interactions	Van der Waals Interactions	Electrostatic Interactions
Native Ligand *	ARG22 ARG90 GLN135 ARG160	LYS28 ARG90 ARG160	__	ALA66, VAL69 TYR70, ASN73 TYR131, TYR138 VAL153, ALA156 TYR157	ARG22, LYS28 LYS87, ARG90 LYS143, ARG160 MG402
NPA004179	ARG22 LYS28 ASN73 GLN135 TYR138 LYS208	__	MET187 LYS191 PRO274	VAL69, TYR70, LYS87, LYS143, VAL152, VAL153, ALA156, TYR157, VAL188, ARG192	__
NPA011911	TYR131 ARG160 LYS191 ARG192 TYR138	__	TYR138 VAL153 ALA156	ALA66, TYR70, ASN73, LYS143, VAL153, ALA156, TYR157, LYS208	LYS28
BIMP003491	LYS28 ARG90 ARG160	__	TYR70 TYR131 TYR138 CYS139 ALA156	ARG22, VAL69, ASN73, LYS87, GLN135, LYS143, VAL153, TYR157	__
BIMP004391	LYS28 TYR138 LYS143	__	VAL69 VAL153 ALA156	ARG22, GLN25, ASN73, ARG90, GLN135, VAL152, TYR157, ARG160	__

* Native ligand (phosphoribosyl pyrophosphate) binding interaction profile was obtained from Uniprot (https://www.uniprot.org/uniprotkb/P9WFR5/entry, accessed on 17 January 2025) and through the 2D interaction diagram of the native ligand bound to Rv3806c (PDB ID: 8J8J) [24].

**Table 2:** Drug-likeliness profiles of the probable four ligands.

ADMET Properties *	Compound ID **			
	BIMP003941	BIMP004391	NPA004179	NPA011911
MW	348.19	360.16	492.2	330.04
nHA	5	6	10	8
nHD	1	2	3	3
TPSA	80.67	93.06	156.66	134.27
LogS	−3.6649	−3.36154	−3.20383	−3.22655
LogP	2.497514	1.206575	1.513767	2.387956
LogD	2.292645	1.463968	1.731872	1.988771
pgp_sub	0.030569	0.434714	0.23759	0.16227
Hia	1.18 × 10^−5^	0.051714	0.29677	0.16819
BBB	0.001444	0.003485	0.00675	0.00021
PPB	71.38541	55.19302	82.976915	80.607352
CYP2D6-inh	2.36 × 10^−6^	1.44 × 10^−9^	1.65 × 10^−7^	8.80 × 10^−6^
CYP3A4-inh	0.036868	0.069623	8.87 × 10^−8^	0.0008331
cl-Plasma	1.797511	3.032425	2.4271849	2.3228227
hERG	0.011908	0.010585	0.03928	0.01306
Ames	0.191869	0.389166	0.330381	0.4483946
Carcinogenicity	0.490848	0.293215	0.1930335	0.4680035
H-HT	0.444384	0.45598	0.4304653	0.3465636

* Compounds were retrieved from the Bioactivity of Indian Medicinal Plants (BIMP) database and the Natural Products Atlas (NPAtlas) database. ** Molecular weight (MW) (≤500), Number of Hydrogen Bond Acceptors (nHA) (≤10), Number of Hydrogen Bond Donors (nHD) (≤5), Total Polar Surface Area (TPSA) (≤140), solubility (LogS) (≥−10, ≤0.5), lipophilicity (LogP) (≤5, ≥−2), pH-dependent lipophilicity (LogD) (≤3, ≥1), protein glycoprotein substrate (pgp_sub) (≤0.5), human intestinal absorption (hia) (≤0.5), blood–brain barrier (BBB) (≤0.5), plasma protein binding (PPB) (≤90), cytochrome isoform CYP2D6 inhibitor (≤0.5; non-inhibitor), cytochrome isoform CYP3A4 inhibitor (≤0.5; non-inhibitor), plasma clearance (cl-Plasma) (>0, ≤15), human-ether-a-go-go-related gene (hERG) (≤0.5), Ames (≤0.5), Carcinogenicity (≤0.5), and human liver toxicity (≤0.5).

**Table 3:** Key determinants supporting the selection of four promising ligands for molecular dynamics simulation analysis.

Compound IDs	Compound Name	2D Structure	Binding Affinities (Kcal/mol)	Interactions with Active Site Residues
BIMP003941	(2R)-2-[(4aR,7S,8R,8aR)-8,8a-dimethyl-7-[(Z)-2-methylbut-2-enoyl] oxy-3-oxo-4,4a,5,6,7,8-hexahydronaphthalen-2-yl] propanoic acid	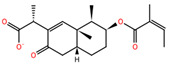	−7.8	15
BIMP004391	(1S,2R,5R,7S,14R,15R,16S,17S)-5,8-dihydroxy-2,9,15,17 tetramethyl-6,13 dioxapentacyclo [12.3.1.05,17.07,16.010,15] octadeca-8,10 diene-4,12-dione	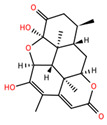	−8.6	12
NPA004179	Penicillactone A	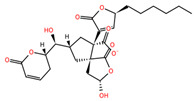	−8.2	13
NPA011911	2,11-dihydroxy-1-methoxycarbonyl-9-carboxyxanthone	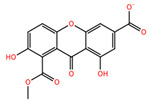	−7.5	13
Native Substrate	5-*O*-phosphono-alpha-d-ribofuranosyl diphosphate	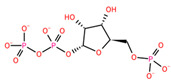	−7.2	17

**Table 4:** Average values of RMSD, RMSF, and Rg obtained through MD trajectories of the protein–ligand complexes.

Complexes	Average RMSD (nm)	Average RMSF (nm)	Average Rg (nm)
Rv3806c–Native Ligand	0.3117 ± 0.034264	0.1002 ± 0.0498	2.0258 ± 0.0170
Rv3806c–NPA004179	0.4431 ± 0.0708	0.1417 ± 0.0864	2.0303 ± 0.0168
Rv3806c–NPA011911	0.3302 ± 0.0472	0.1758 ± 0.0720	2.0479 ± 0.0204
Rv3806c–BIMP003941	0.3842 ± 0.0536	0.1408 ± 0.0629	2.0176 ± 0.0231
Rv3806c–BIMP004391	0.3167 ± 0.0554	0.1473 ± 0.0742	2.0326 ± 0.0237

**Table 5:** Binding free energies (kcal/mol) of Rv3806c–ligand complexes obtained through the MM-PBSA approach, detailing van der Waals, electrostatic, polar solvation, SASA, and total binding energy.

Complexes	ΔG_vdW_	ΔG_elec_	ΔG_polar_	ΔG_SASA_	ΔG_bind_
Rv3806c–Native Ligand	16.75 ± 0.21	−1904.02 ± 1.24	1710.14 ± 1.09	−2.95 ± 0.01	−180.07 ± 0.38
Rv3806c–NPA004179	−52.78 ± 0.1	−37.02 ± 0.24	77.56 ± 0.33	−5.25 ± 0	−17.49 ± 0.2
Rv3806c–NPA011911	−30.3 ± 0.1	−53.58 ± 0.31	61.29 ± 0.22	−3.71 ± 0	−26.3 ± 0.15
Rv3806c–BIMP003941	−36.77 ± 0.07	−2.61 ± 0.19	21.33 ± 0.15	−3.89 ± 0	−21.94 ± 0.12
Rv3806c–BIMP004391	−26.45 ± 0.07	−3.73 ± 0.24	18.06 ± 0.22	−2.8 ± 0.01	−14.91 ± 0.09

## Data Availability

Data is contained within this article and the Supplementary Materials.

## Supplementary Materials

The following supporting information can be downloaded at: https://www.mdpi.com/article/10.3390/ijms27125258/s1.
