# Supplementary material for: Structure-Based Virtual Screening of Natural Product-Derived Inhibitors Targeting Rv3806c in the Decaprenylphosphoryl-d-Arabinose Biosynthetic Pathway of Mycobacterium tuberculosis
Source: Int J Mol Sci. 2026 Jun 10;27(12):5258. doi: 10.3390/ijms27125258 (PMC13299623; doi:10.3390/ijms27125258)
Supplement: Supplementary file 1 [file ijms-27-05258-s001.zip › ijms-4305408-supplementary.pdf]

Title

Supplementary Data

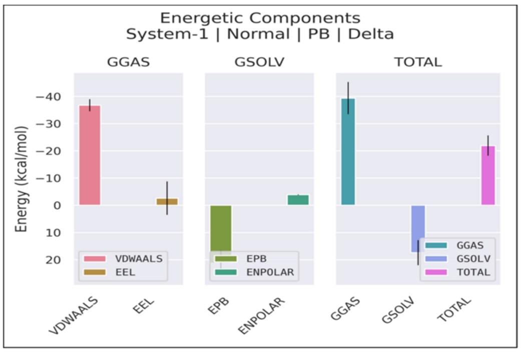

(A) BIMP003941

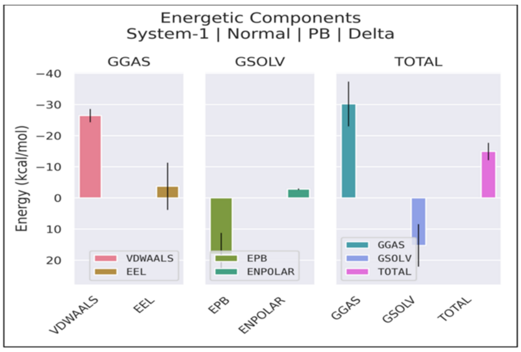

(B) BIMP004391

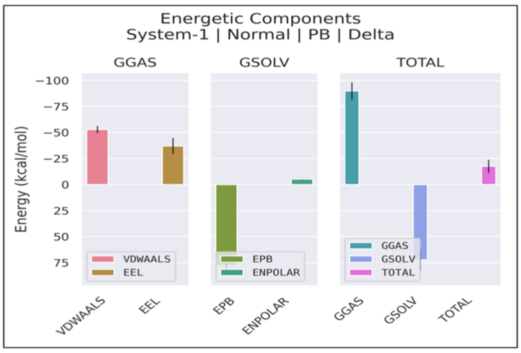

(C) NPA004179

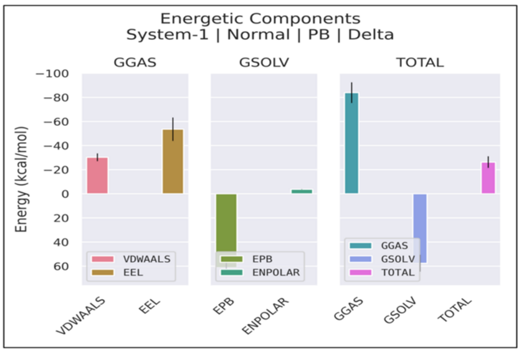

(D) NPA011911

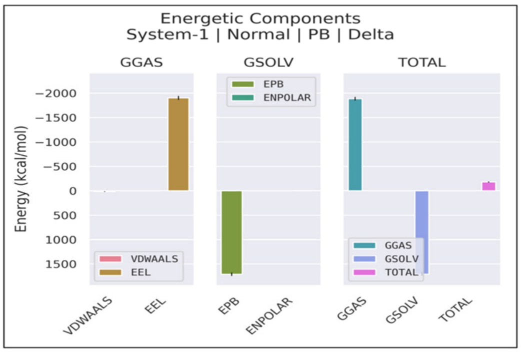

(E) Native Ligand

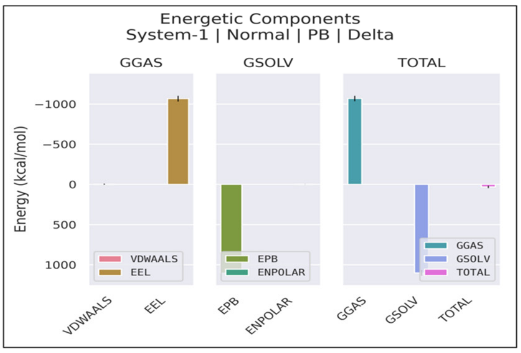

(F) Native Ligand without Mg^{+2}

Figure S1. Binding free energy analysis of the Rv3806c–ligand complexes performed using the gmx_MMPBSA_analyzer tool. The plots illustrate the total binding free energy and its contributing components, including van der Waals, electrostatic, polar solvation, and non-polar solvation energies, throughout the last 10 ns of the respective, MD trajectories.
